# Physiological dynamics of stress contagion

**DOI:** 10.1038/s41598-017-05811-1

**Published:** 2017-07-21

**Authors:** Stephanie J. Dimitroff, Omid Kardan, Elizabeth A. Necka, Jean Decety, Marc G. Berman, Greg J. Norman

**Affiliations:** 1 Department of Psychology, the University of Chicago, Chicago, IL USA; 2 Center for Cognitive and Social Neuroscience, the University of Chicago, Chicago, IL USA; 3 Grossman Institute for Neuroscience, the University of Chicago, Chicago, IL USA; 4 Department of Psychology and the Child Neurosuite, the University of Chicago, Chicago, IL USA

## Abstract

Can viewing others experiencing stress create a “contagious” physiological stress response in the observer? To investigate second-hand stress, we first created a stimulus set of videos, which featured participants speaking under either minimal stress, high stress, or while recovering from stress. We then recruited a second set of participants to watch these videos. All participants (speakers and observers) were monitored via electrocardiogram. Cardiac activity of the observers while watching the videos was then analyzed and compared to that of the speakers. Furthermore, we assessed dispositional levels of empathy in observers to determine how empathy might be related to the degree of stress contagion. Results revealed that depending on the video being viewed, observers experienced differential changes in cardiac activity that were based on the speaker’s stress level. Additionally, this is the first demonstration that individuals high in dispositional empathy experience these physiological changes more quickly.

## Introduction

The ability to share emotional information with one another is an essential part of the human experience; one that not only adds to the richness of life, but is crucial for the coordination of social interactions. Emotional expressions are readily observable by others, for example, through facial expressions, postures, overt behaviors, and fluctuations in vocal tone^1–3^. To successfully navigate the social environment, one must be adept at rapidly reading these emotional cues, and know how and when to appropriately react to them. While this is generally accomplished with little effort, developing a mechanistic understanding of how emotional cues are processed in the receiver is no simple task as it involves the deciphering of dynamically changing facial expressions, postures, gestures, vocal tones and language, in near real time and in the context in which they occur^4^. One such proposed mechanism for the facility of emotional understanding is through emotional contagion, the automatic transmission of emotional states between individuals^5^. The ability to “catch” aspects of another person’s emotions may serve as a relatively fast and effective way of understanding another individual’s affective state, which likely enhances one’s ability to be a successful agent in a highly complex and dynamic social environment^6^. However, individual differences in cognitive and affective empathy create disparities in the ability to accurately understand the meaning of these expressions in others^7^. Because the negatively valenced emotions, and their related physiological correlates, associated with stress have been clearly demonstrated to have deleterious health consequences when present chronically^8^, it is important to understand how these emotions may be transmitted to others. The existence of stress contagion may indicate an additional pathway to these deleterious health consequences^9^.

Emotions, like all psychological processes, are psychophysiological in nature. The physiological response to a stimulus is central to all affective processes^10, 11^. Work by Buchanan *et al*.^12^ was one of the first to demonstrate that the experience of stress may be physiologically “contagious”. In their study, “observers” were panelists in the Trier Social Stress Test (TSST), a task which has been repeatedly shown to elicit a robust stress response in which “speakers” give an impromptu speech to a panel of judges^13^; and markers of stress reactivity (alpha-amylase and cortisol) were measured in both the observers and the speakers. Cortisol release in the observers was proportional to the release in speakers, and amount of alpha amylase and cortisol release was related to empathy levels of the observers. Similar work by Engert *et al*.^14^ had observers watch either a romantic partner or a stranger complete the TSST via a one-way mirror or video. Findings from this study revealed a positive association between cortisol release in the speaker and observer, with the strongest association being between romantic partners via one-way mirror. Similarly, the transmission of negative affect and associated psychophysiological response has been studied in the context of mother and infant interactions, with findings indicating that mothers’ stress responses can impact autonomic reactivity in their infants^15^.

While progress has been made in understanding some of the physiological underpinnings of stress contagion, much remains to be known. Given the health consequences associated with caregiver stress^16^, and the less intense, but more frequent social situations where “empathic stress” or “stress contagion” are likely to occur^17^, more detailed understanding of the social, psychological, and physiological mechanisms that allow for the spread of stress across individuals is likely to have important implications for health. The experience of stress is typically associated with changes in the activity of both the HPA axis and the autonomic nervous system, and different types of stressors can result in different patterns of activation^18, 19^. Under circumstances of intense or chronic stress, both of these stress response pathways have been found to be associated with a variety of unfavorable health outcomes^8^. Thus, while stress contagion has been shown to affect the HPA axis in adults^12, 14^, further research is necessary to better understand how the autonomic nervous system (ANS) reacts in adults. Understanding the extent to which viewing stressed others affects ANS activity in the observer not only provides information regarding potential pathways to later pathology, it also allows for a better understanding of the underlying affective processes due to the increased temporal resolution of ANS responses that cannot be gleaned by monitoring HPA axis activity alone. The HPA axis, being a hormonal system, takes minutes to be engaged, while changes in autonomic activity can be measured on a second-to-second time scale^20^. Determining the latency and magnitude of the ANS response to watching others experiencing stress may provide vital information regarding the particular emotions being induced in the watcher. Moreover, evaluating the co-variation of cardiac responses between the speaker and observer may provide information regarding the ability of the observer to track the emotional state of the individual experiencing stress.

In the current study, we sought to determine individual psychophysiological responses to viewing videos of strangers who are experiencing varied levels of stress, the extent to which this response was associated with that of the participant in the video, and whether this relationship was dependent on levels of empathy in the observer. To do so, we created a stimulus set of videos of people speaking without exposure to a social stressor, during a social stressor, and after experiencing a social stressor, during which we monitored speakers’ cardiac autonomic activity. We then observed the corresponding cardiac responses of participants who viewed these videos. Previous work in adults has solely had participants watch others perform the TSST. While the current study utilized this approach, we also included speakers who were less obviously stressed, in the “Post Stress” videos. Everyday life is rife with these affective puzzles of determining who is stressed when the circumstances make the emotional state of others somewhat ambiguous. Thus, the inclusion of this “Post Stress” group was intended to represent this type of encounter. Furthermore, previous work has not explored the timing of such effects, that is, how long it takes observers to physiologically react to viewing stress. Understanding the time dynamics of these changes is important, as it may reveal inter-individual differences in the way individuals perceive and respond to the emotions of others, which is vital for everyday social functioning. For example, appropriate timing of interpersonal synchrony has been shown to be related to feelings of affiliation for others^21, 22^. We recorded neurocardiac activity on a beat-to-beat basis, and thus were capable of determining physiological co-variation occurring in the speaker and the observer with relatively high temporal resolution. The current study also included many different target speakers, which allowed us to better determine whether these effects were person specific or generalizable across individuals, as previous work has only examined stress contagion from one speaker to one observer.

We hypothesized that observers would show stronger psychophysiological reactions to the “Stress” and “Post Stress” videos as compared to the “No Stress” videos. We also hypothesized that levels of empathy would interact with these physiological responses to viewing others who are stressed, in that empathetic individuals would have greater physiological reactivity in response to viewing stress. Because speed of physiological co-variation might reflect the ability to more quickly predict another’s mental state and then plan an appropriate behavioral response, we also hypothesized that empathetic individuals would be able to achieve co-variation more quickly.

## Results

### Part A

A matched pair t-test revealed the 14 speakers who completed the TSST (speakers in “Stress” and “Post Stress” conditions), experienced a significant decrease in IBI from baseline to TSST, *t*(13) = 8.49, *p* < 0.01, *d* = 1.48 (*M* = 844 ms at baseline to *M* = 653 ms during TSST). Physiological data within each one-minute clip was analyzed in a repeated measures one-way ANOVA and as expected, the percent change in IBI of participants was significantly different based on video type, *F*(2, 18) = 15.51, *p* < 0.01. IBI was lower in “Stress” videos compared to “No Stress” videos, *t*(12) = 6.63, *p* < 0.01, and lower in “Post Stress” videos compared to “No Stress” videos, *t*(12) = 2.76, *p* = 0.03. “Stress” videos had a lower percent change in IBI compared to “Post Stress” videos but the difference did not pass Bonferroni correction, *t*(12) = 2.50, *p* = 0.04.

### Part B

#### Mean IBI

**Figure 1:**
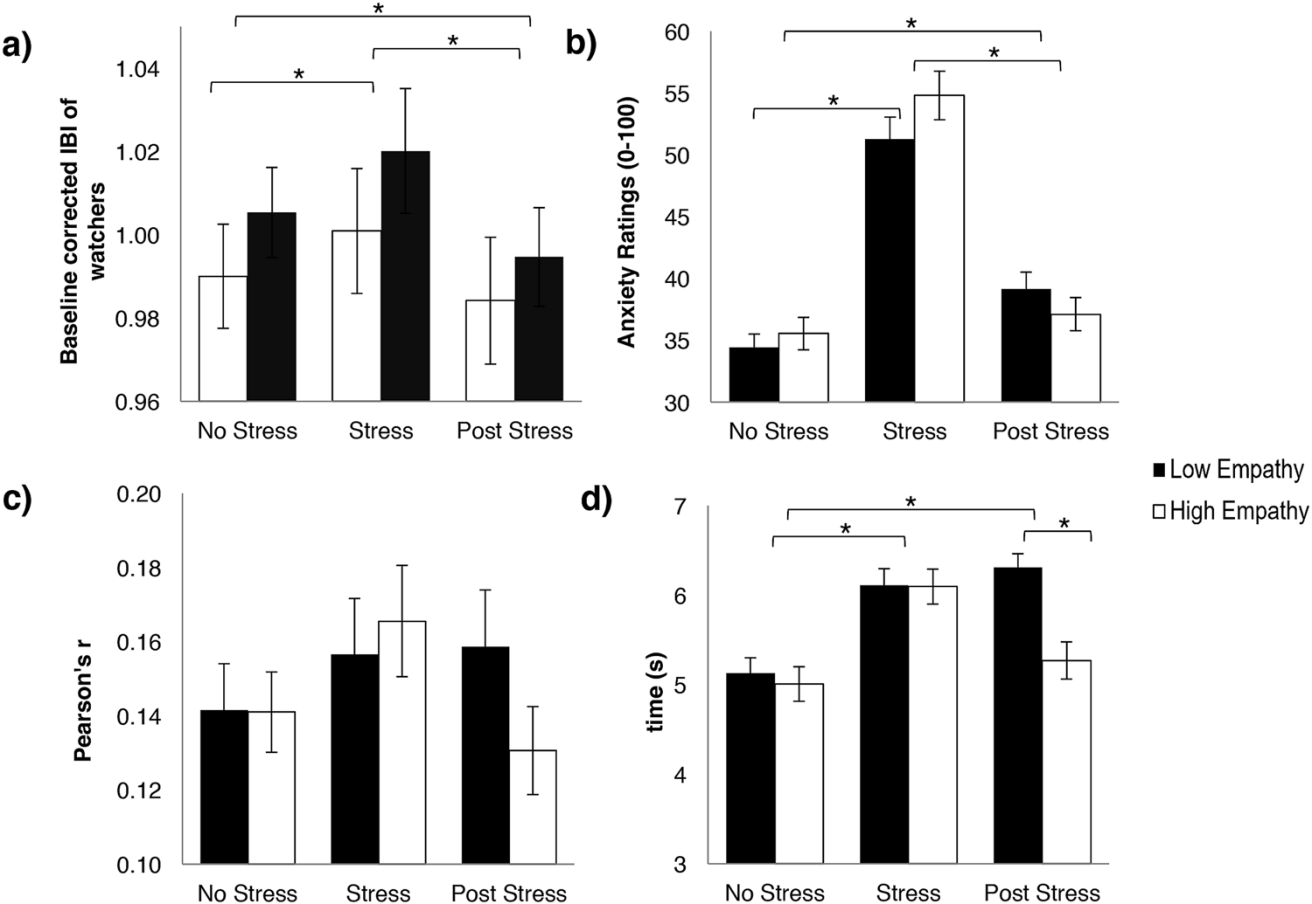
Overview of observers’ responses to viewing videos (**a**) Baseline corrected mean IBI of low and high empathy observers while viewing videos. (**b**) Mean anxiety ratings made by low and high empathy observers while viewing videos. (**c**) Mean maximum time-lagged correlation between IBI of speakers and observers. (**d**) Mean lag (in seconds) to reach maximum correlation between speaker and observer IBI. All error bars represent standard error. *=Statistically significant after Bonferroni correction (*p* < 0.05).

A two-way mixed design ANOVA found no significant interaction between affective empathy and baseline corrected IBI of observers based on video type being viewed, *F*(2, 122) = 1.34, *p* = 0.27. However, as predicted, there was a significant main effect of video type viewed on baseline corrected IBI of observers, *F*(2, 122) = 31.63, *p* < 0.01. IBI was higher when viewing “Stress” videos than when viewing “No Stress” videos, *t*(62) = 4.25, *p* < 0.01, or “Post Stress” videos, *t*(62) = 7.66, *p* < 0.01, and IBI was also higher when viewing “No Stress” videos compared to “Post Stress” videos, *t*(62) = 3.93, *p* < 0.01 (see Fig. 1a).

#### Anxiety Ratings

A two-way mixed design ANOVA found no significant interaction between affective empathy and anxiety ratings made by observers, based on video type, *F*(2, 122) = 1.29, *p* = 0.28. However, as predicted, there was a significant main effect of anxiety ratings based on video type, *F*(2, 122) = 50.05, *p* < 0.01. Speakers in “Stress” videos were rated as significantly more anxious than speakers in “No Stress” videos, *t*(62) = 8.93, *p* < 0.01, and “Post Stress” videos, *t*(62) = 6.12, *p* < 0.01. Speakers in “Post Stress” videos were rated as significantly more anxious than speakers in “No Stress” videos, *t*(62) = 3.75, *p* < 0.01 (see Fig. 1b).

#### Maximum Physiological Correlations and Lags to Maximum

**Figure 2:**
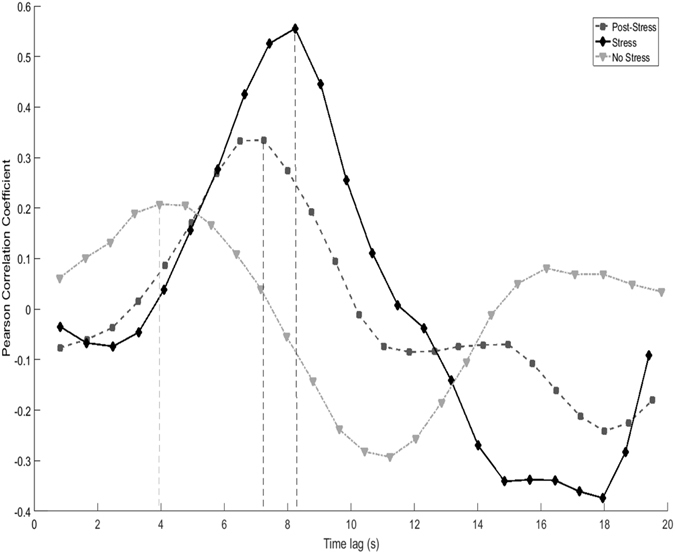
Visual representation of time lag analysis. This data represents one subject. Each line represents the correlation of observer’s IBI with speaker’s IBI, for each video type. As the time lag is increased from zero, we are able to determine at what time the maximum correlation is achieved.

**Figure 3:**
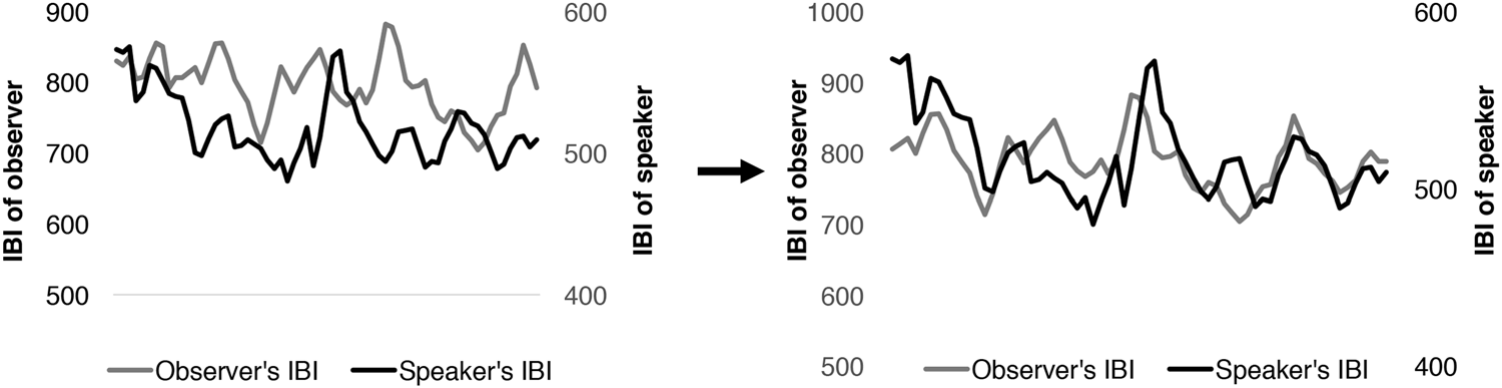
Visual representation of IBI data of observer from Fig. 2 watching a Stress video. The graph on the left shows how IBI of speaker and observer are related when no time lag is implemented. On the left, we see how IBI of speaker and observer are related when a time lag of approximately 8 seconds is applied. A secondary axis was created to help visualize differences in IBI, as the speaker was robustly stressed and as a result had a lower IBI compared to the observer.

A two-way mixed design ANOVA found no significant interaction between affective empathy and maximum correlation between observer and speaker IBI, *F*(2, 122) = 0.86, *p* = 0.43. There was no significant main effect of video type on maximum correlation between speaker and watcher IBI, *F*(2, 122) = 1.17, *p* = 0.31 (see Fig. 1c). However, a two-way mixed design ANOVA revealed a significant interaction between dispositional affective empathy of observers and latency to reach maximum correlation, based on video type, *F*(2, 60) = 3.12, *p* = 0.048 (see Fig. 1d). Lag to reach the maximum correlation between speaker and observer took significantly longer when the observer’s viewing “Post Stress” videos had low, compared to high, affective empathy (5.27 seconds for high empathy observer and 6.31 seconds for low empathy observer), *t*(61) = 2.52, *p* = 0.01. Furthermore, there was a main effect of lag time based on video type, *F*(2, 124) = 10.57, *p* < 0.01. Lag times were shorter for “No Stress” videos compared to both “Stress” videos, *t*(62) = 4.51, *p* < 0.01, and “Post Stress” videos, *t*(62) = 3.14, *p* < 0.01 (See Figs 2 and 3 for visual aid).

Cognitive empathy was not related to any of the independent variables tested. Results including cognitive empathy can be found in the supplemental materials.

## Discussion

The current study sought to determine whether watching individuals under varying levels of stress could induce a “contagious” cardiac response in the observer. The results of this study revealed that observing others experiencing or recovering from stress leads to distinct patterns of cardiac activity in the observer. Past research has shown how being exposed to stressed others can lead to feelings of stress in oneself^17^, and the current study suggests that the ANS may play a role in how stress responses are transferred. While identifying, and reacting to stress in others is certainly important for sustaining relationships^23^ its deleterious influences on health outcomes is still unknown.

As predicted, the results demonstrated that observers’ physiological activity changed in response to the level of stress in the speakers (Fig. 1a). Autonomic activity remained at baseline when viewing others who were not experiencing stress, yet diverged when viewing speakers who were stressed or recovering from stress. Interestingly, the direction of autonomic change in observers when viewing stress and stress recovery was opposing. When viewing speakers who were recovering from stress, we found the expected decrease in IBI from baseline in observers, which is consistent with the most simplified models of stress contagion that assume the directionality of the stress response is conserved. However, viewing others who were overtly stressed induced an autonomic change in the opposite direction, reflecting cardiac deceleration. Previous literature has demonstrated that, depending on the context of a stressor, cardiac deceleration may be indicative of a ‘freezing’ stress response^24^. Cardiac deceleration responses generally occur in situations when no behavioral response is necessary or during periods of information collection^25, 26^. Passive stressors, like viewing aversive images or videos, have been shown to elicit cardiac deceleration^24, 27^. Thus, while watching “Stress” videos created a physiological change that was in the opposite direction of that in the speakers, this change may still be indicative of a stress response. Indeed, the videos contained aversive content (e.g. watching someone defend themselves from a cheating allegation), and required no action from the observer, thus the context of this “stressor” resulted in cardiac deceleration in the observers. This highlights the complexity of stress contagion, and demonstrates that the stress response of the observer is not necessarily identical to the response of the speaker, and instead, is likely dependent on contextual factors associated with the situation. Future work will be necessary to understand the details of how context can change how stress and the corresponding emotions are physiologically represented when they are transmitted from one individual to the next.

In addition to experiencing physiological changes in response to the video set, observers were also able to accurately judge levels of stress in the speakers. Notably, observers were also capable of differentiating speakers who were in the “No Stress” condition from speakers who were recovering from stress. Importantly, these videos were matched in content, yet the stress-recovery videos ostensibly contain stress cues that allow observers to accurately discern levels of anxiety in the speaker. Future work must be conducted to determine which cues are being used, and to what degree they are being used to influence judgments of anxiety.

When analyzing IBI linkage between speakers and observers on a second-to-second level, we observed that the magnitude of the linkage did not differ as a function of the type of video viewed. This analysis was done using a time lag, to determine when in time the maximum correlation between IBI of speaker and observer occurred. Given the importance of inter-personal synchrony in social interactions, these results add to the notion that timing of physiological changes may also play a key role in healthy social functioning, and provide an additional measure that can be investigated in the context of psychophysiology and emotion. Interestingly, there were differences observed in lag time for type of video viewed; with “Stress” videos inciting the longest latency and “No Stress” videos having the shortest latency. Physiological linkage may have taken the most amount of time when watching “Stress” videos because of the complexity of the emotional content, while “No Stress” videos may have taken the least amount of time because of the lack of strong affective cues.

We initially hypothesized that highly empathetic individuals would have greater physiological reactivity in response to viewing stress. We failed to find this effect, as physiological reactivity to viewing stress did not differ in observers with either low or high levels of empathy. In contrast, we observed that high affective empathy was related to a decreased latency to achieve physiological linkage with speakers who were recovering from stress. This is the first demonstration that increased levels of affective empathy are related to a decreased latency to be affected by the emotional state of another individual, however the directionality of this relationship is not currently understood. Future work should explore this avenue of investigation to determine what the social implications are for faster psychophysiological reactivity in response to others. Given how quickly the complex constellation of emotions of a social interaction may change, recognizing and responding to such changes in a timely manner may shape the course and success of a social exchange.

Limitations of the current data include the sample that was used, which was mainly composed of undergraduates at the University of Chicago, and thus we do not know if these results would generalize to a more representative sampling of the population. Furthermore, this work was done via video, and thus the ecological validity of these results in relation to in-person social communication will need to be explored. Given Engert *et al*.’s^14^ findings that suggest stress contagion is stronger via one-way mirror as compared to video, our findings may be amplified should they be tested in an in-person setting. Finally, while our results do demonstrate physiological changes in response to stress of others, we cannot draw any health implications from our specific set of data, as we relied on IBI as our biological measure, and our study was designed to measure acute changes, not long-lasting ones. IBI is directly influenced by both the sympathetic nervous system (SNS) and the parasympathetic nervous system (PNS). An important next step in this area of research is to examine the dual influence of the SNS and PNS in relation to stress contagion, to determine which system is primarily responsible for changes in IBI in response to stressed others. Low heart rate variability (a measure of PNS activity) has been shown to be a marker of all-cause mortality, thus we believe it especially important to examine whether the PNS plays a role in stress contagion, and if it does, whether these changes in PNS can be long-lasting. Future work should measure SNS and PNS concurrently, and consider employing a longitudinal design to determine whether these effects can be persistent in an individual if one is chronically exposed to stressed others.

These data add to the existing literature of emotional contagion research, and bolster the idea that stress can be contagious on a psychophysiological level, albeit in a more complex way than previously recognized. These particular findings are of importance as they demonstrate that individuals can detect stress in others, even in the absence of overt context-dependent stress cues (i.e., stressful topic of speech), and have cardiac responses that are related to those of the speaker. Furthermore, these results elucidate the timing of such effects and how they are related to individual differences in affective empathy. Future research will be necessary to further investigate the mechanisms behind these effects, for example, which modalities are most important in the transmission of stress from one person to another.

This line of research warrants future study. Reacting to another’s affective state is an important factor in forging and maintaining social connections^28^, and future work in this direction may help us gain insights into its psychophysiological underpinnings. Furthermore, studies on the autonomic nervous system’s role in stress contagion will be important in determining whether  second-hand stress may have similar health consequences to first-hand stress^20^. Understanding others is the basis for our lives as social beings, and emotional contagion research helps to inform our knowledge of the transmission and reception of affective states between two individuals.

## Methods

### Stimulus Set Generation

#### Participant Speakers

Twenty-one participants (11 females, 18–22 years old, *M* = 19.65, *SD* = 1.18, 12 Caucasian) who were TSST-naïve were selected from a larger set of participants to be included in the stimulus set of videos. Participants were chosen out of a larger group of speakers based on a number of selection criteria (see section *video generation*). All participants provided their written informed consent as approved by the University of Chicago Institutional Review Board.

#### Procedure

The study was approved by the University of Chicago Institutional Review Board, and the methods were carried out in accordance with approved guidelines. Participant speakers completed a number of self-reported measures, including the Questionnaire of Cognitive and Affective Empathy (QCAE^29^), which is the primary focus of the current manuscript. Once completed, participant speakers were fitted with surface electrodes for the measurement of the electrocardiogram. Participant speakers were then given a standard grey shirt to wear and instructed to sit in a chair that was in front of a wall draped with a white sheet. E-Prime 2.0 (Psychology Software Tools, Pittsburgh, PA) was used to display experimental instructions on a 39” LED TV facing the participants. A high-resolution webcam (Logitech HD Pro Webcam C920) was fitted above the TV facing the participant, and a microphone was installed on a table next to them (Audio-Technica ATR2500-USD Cardioid Condenser USD Microphone). Once seated, the video recording of participant speakers began. The first section of the experimental task was a five-minute long rest period to obtain the participants’ physiological baseline. Following the rest period, individuals completed a series of tasks, some of which included a neutral speech (No Stress), TSST (Stress) or a post-stress neutral speech (Post Stress).

Participants who completed a neutral speech were given a prompt (either to talk about their morning routine or to give a detailed description of the interior of their house), were told they had two minutes to think of what they would say, and then were instructed to speak for three minutes.

Participants who completed a TSST were told that they were accused of cheating on the GRE and had to defend themselves, were given two minutes to prepare a speech, and then spoke for three minutes while the experimenter watched with a neutral expression taking notes. As is standard for this stress induction paradigm^13^, participants were told behavioral analysts would judge a video of their speech on intelligibility, clarity and quality of content.

Participants who completed a “Post Stress” speech first performed a TSST, followed by a ten-minute rest period, followed by a neutral speech describing either their morning routine or the interior of their house. Their neutral speech, which was given during a state of stress recovery, was the one used for the experiment.

#### Video Generation

From the set of videos collected (No Stress, Stress, Post Stress), videos of 21 participants were edited and selected to be part of the stimulus set for the second part of the study (referred to as Part B). All videos were edited using AVS Video Editing software. Videos were framed so that only participants’ faces and their bodies from the elbows up were visible. Each video was edited down to one minute in duration. In all, the 21 videos were comprised of seven “No Stress” videos, seven “Stress” videos and seven videos of “Post Stress”. We ensured each video featured a different participant. Physiological data were analyzed with HRV Analysis 3.1.0 (Mindware, Gahanna, Ohio). Videos were chosen from the larger stimulus set based on a number of criteria. Videos with any audiovisual aberrations, or unclear speech were excluded. Speakers whose ECGs revealed any abnormal recurrent ecoptic heartbeats were also excluded. Once the aforementioned videos were excluded, videos were chosen based upon cardiac responses. Both “Stress” and “Post Stress” videos were comprised of the seven speakers who exhibited the strongest cardiac responses during their respective experimental phase, while ensuring a balanced number of males and females were selected. “No Stress” videos were comprised of the seven speakers who remained closest to their physiological baseline, while ensuring selected videos created a balanced gender distribution. The videos were randomly ordered and compiled into an E-Prime script. Two different orderings were made. In each E-Prime script, there were three blocks, each composed of seven videos of differing types.

#### Participant Observers (Part B)

Seventy-one participants were recruited to be observers. Eight participants were excluded due to repeated movement artifacts in their ECG signal or not following instructions. Sixty-three participants (41 female, *M* = 20.50 years old, *SD* = 2.684, 55.56% Caucasian) were included in the data analysis as observers. All participants provided their written informed consent as approved by the University of Chicago Institutional Review Board. Participants were instructed to refrain from consuming caffeine or participating in strenuous activity for two hours prior to participation.

#### Procedure

The study was approved by the University of Chicago Institutional Review Board, and the methods were carried out in accordance with approved guidelines. Observers first completed the same questionnaires that were administered to participants who were part of the stimulus set. Once fitted with surface electrodes participants were seated in front of a 39^″^ LED TV. Before the start of the videos, participants sat quietly for collection of a five-minute physiological baseline. Participants then watched the 21 videos embedded into the E-Prime script described above in “Video Generation.” Following each video, a pop-up screen asked: “How anxious was the person in the video”? where participants indicated their answers on a visual analog scale with a mouse. Between each video there was a 10 second fixation cross, and between each block of seven videos there was a three-minute break.

#### Autonomic measures

A standard lead II configuration was used for obtaining the electrocardiogram (ECG) on both speakers and observers. Data were collected using a BioNex two-slot mainframe (Mindware Technology, Gahanna, OH) which was connected to a personal computer. The sampling rate of the electrocardiogram (ECG) signal was 1000 Hz. Analysis of the ECG signal was performed using Mindware Technology’s HRV software, Version 3.10. Visual inspection and manual editing of the data was completed to ensure proper removal of artifacts and ectopic beats^30^. The inter-beat interval series was time sampled at 4 Hz to obtain an equal interval time series. Results are described in inter-beat interval (IBI) of the heart, which represents the time in milliseconds between two heartbeats, thus as heart rate increases, IBI decreases.

#### Measure of Empathy

Dispositional empathy in observers was assessed with the Questionnaire of Cognitive and Affective Empathy (QCAE^29^), a standardized method of obtaining trait levels of both cognitive and affective empathy. Dividing observers into low and high empathy groups via a median split preceded all statistical tests performed.

#### Video type and observer physiological activity

To calculate average inter-beat interval (IBI) for each video type, software was used to determine the average IBI of each observer during each video viewed. To calculate the average IBI of each video type, the 7 IBI values for each video type were averaged. All IBIs were baseline corrected using each individual observers’ first five minutes of baseline. Thus, five IBI values, representing the average IBIs for the first five minutes of baseline, were averaged and used as the denominator to create baseline corrected IBI values. A two-way mixed design (empathy x video type) ANOVA was performed using these values, dividing observers into low and high empathy groups via a median split on empathy scores. All multiple comparisons performed were Bonferroni corrected at *p* < 0.05.

#### Anxiety Ratings

Anxiety ratings were made on a visual analog scale by observers, which were converted into numerical scores that ranged from zero to 100. To calculate the average anxiety score of each video type, the seven anxiety scores for each video type were averaged. A two-factor mixed design ANOVA, dividing observers into low and high empathy groups. All multiple comparisons were Bonferroni corrected.

#### Time series analysis

For each video, a time series analysis was performed using the IBI of the speaker and the observer. This was done by correlating the IBI signal from the speaker and *lagged* IBI signal of the observer, for lags between 0 to 20 seconds. By lagged signal, we refer to one where the first *k* (*k* between 0 to 20 seconds) data points of the signal (the IBI values) are discarded, and the rest of the signal is shifted backwards by the same amount in time. The process is akin to holding one signal (speaker’s IBI) untouched, while sliding the other signal (observer’s IBI) backwards in time in steps of one beat, and calculating the Pearson correlation between the speaker’s signal and the lagged signal from the observer at each lag as it increases from 0 seconds up to 20 seconds. Twenty seconds was chosen as the maximum lag in order to maximize our potential to quantify longer latencies, while keeping the data analytic approach practical. The correlation between the two signals typically increases as the lag gets longer up to an optimal lag, and then begins decreasing again when the introduced lag gets too long. We shifted the speaker’s signal by the lag because we expected the physiological response from the cause of the contagion (speaker) to temporally precede physiological response of the observer. That optimal lag where the speaker’s and the observer’s IBI signals are correlated maximally, as well as the size of the correlation at the optimal lag were compared to the zero-lag correlation (zero-lag correlation is simply the Pearson’s correlation between the IBI signals of speaker and observer with no lag, which is used to adjust the correlations at other lags as a baseline correction), were then quantified for each observer and video. The sliding (lagging and correlating) of signals analyses were performed using custom MATLAB R2014a (The MathWorks, Natick, MA, USA) scripts. The optimal lags associated with the maximum correlation between signals were calculated using the MATLAB function ‘findpeaks’, where the criteria for the correlation peak were that it had to be largest over all of the lags, as well as least 0.01 larger than its values at the previous and the next lag. For every combination of speaker and observer, a maximum correlation (Pearson’s *r*) was found, as well as the lag (seconds) at which this maximum correlation occurred.

## Electronic supplementary material

Physiological dynamics of stress contagion - Supplemental
