# Supplementary material for: Physiological dynamics of stress contagion
Source: Sci Rep. 2017 Jul 21;7:6168. doi: 10.1038/s41598-017-05811-1 (PMC5522461; doi:10.1038/s41598-017-05811-1)
Supplement: Supplementary file 1 — Physiological dynamics of stress contagion - Supplemental [file 41598_2017_5811_MOESM1_ESM.pdf]

Physiological Dynamics of Stress Contagion

Stephanie J. Dimitroff [a*], Omid Kardan [a], Elizabeth A. Necka [a], Jean Decety [a,d], Marc G. Berman [a,b,c], Greg J. Norman [a,b,c]

[a]Department of Psychology, the University of Chicago, Chicago, IL

[b]Center for Cognitive and Social Neuroscience, the University of Chicago, Chicago, IL

[c]Grossman Institute for Neuroscience, the University of Chicago, Chicago, IL

[d] Department of Psychology and the Child Neurosuite, the University of Chicago, Chicago, IL

**Corresponding author:*

Stephanie J. Dimitroff, Department of Psychology, 5848 S University Ave, University of Chicago, Chicago, IL 60637, tel: 872-803-3341 sdimitroff@uchicago.edu

Supplemental

Video Generation

The 21 videos were chosen out of a larger subset of 54 videos. Firstly, videos were chosen based on clarity of the cardiovascular data, ensuring that clear signal was obtained for every heartbeat. Secondly, we chose videos based on physiological responses (e.g. "Stress" and "Post Stress" videos were composed of the highest physiological responders (while ensuring a gender balance) and "No Stress" videos were composed of speakers who exhibited the smallest physiological changes during speech.

Videos were then edited from three minutes in duration to precisely one minute in duration. Four of the 21 videos were edited to start two to four seconds from time zero, in order to coincide with the onset of speaker's speech (and avoid 2-4 seconds of silence at the beginning of the video). Five of the seven "No Stress' videos were edited to feature either the second or third minute of speech because these time frames featured the speaker while their HR was closest to baseline levels.

Results

A two-way mixed design ANOVA found no significant interaction between cognitive empathy and baseline corrected IBI of watcher based on video type being viewed, $F(2, 122) = 0.54$, $p = 0.59$.

A two-way mixed design ANOVA found no significant interaction between cognitive empathy and anxiety ratings made by observers, based on video type, $F(2, 122) = 2.373$, $p = 0.098$.

A two-way mixed design ANOVA found no significant interaction between cognitive empathy and maximum correlation based on video type, $F(2, 122) = 0.71$, $p = 0.49$.

A two-way mixed design ANOVA found no significant interaction between cognitive empathy and lag time to obtain maximum correlation based on video type, $F(2, 122) = 0.56$, $p = 0.57$.
